# Clinical trajectories of individuals with severe mental illness continuing and discontinuing long-acting antipsychotics: a one-year mirror-image analysis from the STAR Network Depot study

**DOI:** 10.1038/s41537-023-00342-3

**Published:** 2023-04-17

**Authors:** Giovanni Ostuzzi, Federico Tedeschi, Federico Bertolini, Carlo Cotugno, Andrea Aguglia, Francesco Bartoli, Giuseppe Carrà, Armando D’Agostino, Giovanni Martinotti, Corrado Barbui, Chiara Gastaldon, Davide Papola

**Affiliations:** 1 WHO Collaborating Centre for Research and Training in Mental Health and Service Evaluation, Department of Neuroscience, Biomedicine and Movement Sciences, Section of Psychiatry, University of Verona, Verona, Italy; 2 Hospital “Villa Santa Giuliana”, Verona, Italy; 3 Department of Neuroscience, Rehabilitation, Ophthalmology, Genetics, Maternal and Child Health, Section of Psychiatry, University of Genoa, Genoa, Italy; 4 Istituto di Ricovero e Cura a Carattere Scientifico (IRCCS), Ospedale Policlinico San Martino, Genoa, Italy; 5 Department of Medicine and Surgery, University of Milano-Bicocca, Monza, Italy; 6 Division of Psychiatry, University College London, London, UK; 7 Department of Health Sciences, University of Milan, Milan, Italy; 8 Department of Neurosciences, Imaging and Clinical Sciences, Università degli Studi G. D’Annunzio, Chieti-Pescara, Italy

**Keywords:** Psychosis, Schizophrenia

## Abstract

Evidence on long-acting antipsychotics (LAIs) in unselected populations with severe mental illness is scant. In this mirror-image study, we compared multiple clinical outcomes 1 year before and after a first LAI prescription in adults with severe mental illness, describing clinical trajectories of LAI continuers and discontinuers. We compared LAI continuers and discontinuers through Mann–Whitney *U* test, Kaplan–Meier survival curves, regression for interval-censored data, and a maximum-likelihood mixed-model with individual random-effect and time as predictor. Of the 261 participants analyzed, 71.3% had schizophrenia-spectrum disorders, and 29.5% discontinued the LAI before 1 year. At baseline, LAI discontinuers had a shorter illness duration, lower attitude and adherence scores. The mirror-image analysis showed reduced hospital admissions only for LAI continuers. Over time, continuers spent less days hospitalized, but had more adverse events and more antipsychotics prescribed, with higher overall doses. In conclusion, this study shows that LAIs might be beneficial in unselected patient populations, provided that adherence is maintained. LAI continuers spent less time hospitalized, but received more antipsychotics and suffered from more cumulative adverse events over time. Therefore, the choice of initiating and maintaining a LAI should be carefully weighed on a case-by-case basis.

## Introduction

Treatment adherence is a major issue for many severe mental disorders, as it has been associated with clinical relapse, re-hospitalization, and functional deterioration^1,2^. Long-acting antipsychotics (LAI) are at least as effective and tolerable as oral antipsychotics for the prevention of relapse in people with schizophrenia-spectrum disorders according to both randomized^3,4^ and observational studies^5^. Many researchers argued that earlier and broader employment of LAIs might improve individual long-term outcomes, adherence, overall acceptability of treatments^1,6,7^, and even help overcoming global mental health issues^8^. In line with this reasoning, paliperidone 1-monthly and risperidone LAI have been recently included in the WHO Essential Medicine List, aiming to expand their availability in middle-income countries and constrained-resource settings^9^.

Available evidence on LAIs is largely focused on people with schizophrenia-spectrum disorders, although in routine clinical practice these formulations are often prescribed off-label for other severe mental disorders, particularly bipolar and personality disorders, for which clinical evidence of efficacy and tolerability is relatively scant^10–13^.

Moreover, the real-world applicability of randomized trial findings has been questioned, due to their distance from clinical populations, outcomes and practices^14,15^. Observational studies could help to fill this gap and include the assessment of participant-centered outcomes, such as quality of life and attitude towards treatments, which remain understudied. Many previous observational studies compared clinical outcomes (e.g., number of relapses or hospital admissions) before and after the introduction of LAIs, using both pre-post and mirror-image approaches^5^. In these studies, however, those who discontinued the LAI during follow-up were either removed from the analysis or pooled with continuers^16^, which is likely to bias the true estimate of the effect of LAIs compared to previous treatments (i.e., oral antipsychotics).

On these premises, we conducted an observational, mirror-image study in individuals with various psychiatric disorders followed under ordinary clinical practice, comparing hospitalization, psychopathology, use of psychotropic medications, and adherence/attitude towards treatments during one year before and after the first LAI prescription.

## Methods

The STAR Network (*Servizi Territoriali Associati per la Ricerca*—Community Services Associated for Research) is a consortium of clinicians and researchers working in Community Psychiatric Services across Italy, aimed at collecting original data from real-world clinical practice^17–19^. The STAR Network “Depot” Study is an observational study conducted in Italy between 2015 and 2018^13,20,21^. This study was conducted independently from industry funding or support. The study protocol was approved by the Local Ethics Committee of the coordinating center (Ethics Committee for Clinical Trials of the Provinces of Verona and Rovigo, protocol no. 57622 of the 09/12/2015) and of each of the 36 participating centers, and was made publicly available on the online repository Open Science Framework (https://osf.io/wt8kx/). The present study was drawn up following the ‘STrengthening the Reporting of OBservational studies in Epidemiology’ (STROBE) Statement items^22^.

### Study design and population

We consecutively recruited individuals prescribed with any LAI antipsychotic over a period of 12 months, and assessed them after 6 and 12 months. Participants were eligible for the study if they were: (a) 18 years old or older; (b) willing to sign the informed consent; (c) beginning a LAI medication; (d) with no LAI use over the previous three months. The simultaneous prescription of other medications, including oral antipsychotics, was not an exclusion criterion. We included participants from different settings, including outpatient facilities, hospital psychiatric wards, daytime community centers, and residential facilities. For the aims of this mirror-image analysis, we further selected individuals based on the following criteria:

- individuals diagnosed with one of the following psychiatric disorders: schizophrenia spectrum (ICD-11 codes 6A20 to 6A24), bipolar disorders (ICD-11 codes 6A60 to 6A6z), and personality disorders (ICD-11 code 6D10);
- LAI-naive individuals (receiving a LAI prescription for the first time in their life);
- individuals treated with oral antipsychotics over the previous 12 months;
- individuals with 12 months follow-up data available.

We considered five clinically relevant outcomes:

- Hospitalization (including number of hospital admission and number of days hospitalized);
- Psychopathology (measured as a rating scale score);
- Medications’ cumulative dose (i.e., cumulative number and dose of both antipsychotics and all prescribed psychotropic medications);
- Adherence and attitude towards medications, as perceived by both clinicians and participants;
- Adverse events (number of adverse events reported by participants).

We analyzed hospitalization data according to a mirror-image approach, comparing the number of hospital admission and number of days hospitalized the year before with the year after the introduction of the LAI medication. For the remaining outcomes, we used a pre-post approach, comparing the same population before and after the introduction of LAIs.

The analysis was conducted on the overall eligible population and on two subgroups of individuals, indicated as “continuers” and “discontinuers”. We defined “continuers” those participants receiving a LAI medication over the entire 12-month follow-up, irrespective of whether the LAI prescribed at baseline was continued for 12 months or one or more switches to another LAI occurred during the follow-up period. In case of switch to another LAI, we considered “continuers” only those for whom less than 2 months elapsed from the last administration of the previous LAI to the first administration of the next LAI. We defined as “discontinuers” all remaining participants, who interrupted the prescribed LAI during follow-up.

Socio-demographic and clinical data were collected at baseline and after 6 and 12 months by means of the following tools:

- Recruitment and follow-up forms, which included socio-demographic, clinical and pharmacological information;
- The clinician-rated Brief Psychiatric Rating Scale (BPRS)^23^, validated in Italian^24^, which assesses the overall level of psychiatric symptoms. Scores ranging from 31 to 40 indicate mild symptoms, from 41 to 52 moderate symptoms, and above 52 severe symptoms^25^. According to Shafer, five subscales can be described (affect, positive symptoms, negative symptoms, resistance, and activation);^26^
- The self-administered Drug Attitude Inventory 10-items (DAI-10)^27^, validated in Italian^28^, which measures attitudes toward medications. The scores range between −10 and 10, with higher scores indicating a better overall attitude toward medications;
- The clinician-rated Kemp’s 7-point scale^29^, compiled by the clinician, which assesses overall adherence to treatments. The scores range from 1 to 7, with higher scores indicating equally higher levels of adherence. Scores of 5 and above indicate an overall good acceptance of medications.

### Data management and statistical analysis

Recruiting centers periodically forwarded baseline and follow-up data to the coordinating center (University of Verona), which archived and entered them into a computerized database. Data correctness and consistency was ensured by a double-entry technique and by a set of electronic and manual edit checks. Participants’ data were recorded anonymously. A unique number both in the recruitment and follow-up forms and in the database identified participants. Total confidentiality of data was guaranteed throughout the entire course of the study, in accordance with the Declaration of Helsinki^30^.

The number and percentage of discontinuers were calculated, and descriptive statistics were used to describe the main epidemiological and clinical characteristics of both the global recruited population, and of continuers and discontinuers separately. We collected the following variables of interest: illness duration and age at onset (in all cases, both as a continuous variable, and as a categorical one, by dividing it into the following age groups: 18–30, 31–45, 46–64, 65+), gender, citizenship (Italian vs other), housing condition (alone, with partner and/or children, with other relatives, any residential home), marital status (non-conjugated vs conjugated), educational level (illiterate/no title, primary school, lower-secondary school, diploma, university degree), working status (employed, unemployed, student, retired, housewife/other), diagnosis (schizophrenia spectrum, bipolar disorder, personality disorders) and LAI type (first-generation, risperidone, paliperidone, aripiprazole, olanzapine). Continuous variables were expressed as means and standard deviations, while categorical variables were expressed as absolute numbers and percentages. Continuer and discontinuer values at baseline were statistically compared through Mann–Whitney *U* test in the case of continuous variables, and through Chi-squared test in case of categorical variables. Descriptive statistics on reason for withdrawing (for discontinuers) were also computed.

We performed survival analyses by considering continuers as censored at 12 months (see the Supplementary Information for details on the definition of treatment duration). We drew Kaplan–Meier survival curves (with their confidence intervals) for the whole sample and for each diagnostic group separately. Then, we performed a regression for interval-censored data on the whole sample^31^, using diagnostic group as the only predictor (details are given in the Supplementary Information).

The number of hospital days was divided into three groups (zero, up to 14 days, more than 14 days) and considered as a categorical ordinal variable. The Brant test^32^ was performed in order to assess whether performing a multilevel ordered logit or multinomial logit regression: first, we run a model with only the time indicator as predictor to compare baseline and follow-up with respect to their distribution in these three categories (both for the whole sample and continuers and discontinuers separately), then in a model with also an indicator variable for being a continuer and its interaction with time in order to compare change between the two time points. In both cases, we used the Stata package *gllamm*^33^ to perform Brant test. For each interval-level clinical variable, in order to fully exploit information from both baseline and 12-month-follow-up values, mean values of interval-level clinical variables (together with the standard error of their estimate) at both time points were jointly estimated through maximum-likelihood with missing values. A maximum-likelihood mixed-model with individual random-effect and the time indicator as the only predictor was performed to assess presence of a change between baseline and follow-up. Such models allowed for a non-null correlation and distinct variances between baseline and 12-month values, and were estimated both considering the whole sample and the two subgroups (continuers and discontinuers) separately. The mixed-model analysis was then repeated including in the equation an indicator variable for being a continuer and its interaction with the time indicator, to assess whether the change between baseline and 12 months was associated with being a continuer. In the analyses on interval-level variables, robust standard errors were adopted to allow for heterogeneity at the individual level. As a sensitivity analysis, such models were repeated for the subgroup of participants with schizophrenia-spectrum disorders. Statistical analyses were performed with the software Stata 17^34^.

## Results

### Characteristics of the sample

**Fig. 1:**
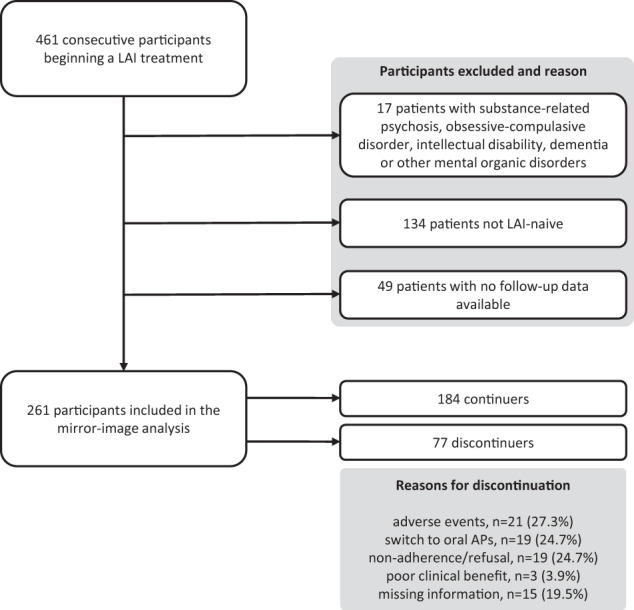
Selection of patients for the mirror-image analysis. AP antipsychotic, LAI long-acting injectable antipsychotic.

Of the 416 originally recruited participants, 261 participants (51.9%) were eligible for the mirror-image analysis, as they were LAI-naive, had a diagnosis of schizophrenia-spectrum disorders, bipolar disorder or personality disorders, and provided data at 12 months of follow-up (Fig. 1). Overall, the mean age of participants was 41.4 years (standard deviation (sd) 13.4) and they were mostly men (59%) and Italian (87.7%). We found relatively low levels of social support and social/working functioning, as 52.9% lived with parents/relatives and 3.8% lived in residential homes; only 14.2% were married, and 53.6% were unemployed. Most participants had either a lower-secondary (44.0%) or a high-school degree (39.3%). Schizophrenia-spectrum disorders were the most common diagnoses (namely, 55.2% had a diagnosis of schizophrenia and 16.1% of schizoaffective disorders), followed by bipolar disorder (21.8%) and personality disorders (6.9% on the total, of which 38.9% had borderline personality disorder, 27.8% schizoid personality disorder, and 33.3% mixed or unspecified personality disorders). The onset of disease was before 18 years for 10% of participants, while for the majority of them (49.4%) it was between 18 and 30 years. Further, 56.7% of participants received a diagnosis at least 6 years before the time of recruitment. The most frequently prescribed LAIs were paliperidone palmitate one-monthly and aripiprazole LAI (28.4% in both cases), and the group of first-generation LAIs (28.0%, of which the most represented was haloperidol, prescribed in 18.4% of participants). Socio-demographic and clinical characteristics at baseline were generally similar between continuers and discontinuers, with the exception of significantly higher scores for continuers on both the Kemp scale (5.04 vs. 4.45, *p* = 0.004) and the DAI-10 (2.88 vs. 0.83, *p* = 0.003), and significantly shorter illness duration in discontinuers (with a higher percentage of people with illness duration below 1 year, namely 36% vs 12%, *p* < 0.001) (Table 1).

**Table 1:** Baseline characteristics of participants continuing and discontinuing a first LAI prescription over a follow-up period of 12 months.

Variables	All patients	Continuers	Discontinuers	*p* value
Age, mean (sd)	41.4 (13.4)	40.8 (13.1)	42.8 (14.0)	0.263
Age categories, *n* (%)				0.554
18–30	72 (27.6)	50 (27.2)	22 (28.6)	
31–45	84 (32.2)	64 (34.8)	20 (26.0)	
46–60	84 (32.2)	56 (30.4)	28 (36.4)	
≥61	21 (8.0)	14 (7.6)	7 (9.1)	
Female, *n* (%)	107 (41.0)	71 (38.8)	36 (46.2)	0.221
Italian, *n* (%)	228 (87.7)	165 (90.2)	63 (81.8)	0.061
Housing conditions, *n* (%)				0.439
Alone	63 (24.1)	41 (22.3)	22 (28.6)	
With partner and/or children	50 (19.2)	34 (18.5)	16 (20.8)	
With other relatives	138 (52.9)	103 (56.0)	35 (45.5)	
Any residential home	10 (3.8)	6 (3.3)	4 (5.2)	
Conjugated, *n* (%)	37 (14.2)	26 (14.1)	11 (14.3)	0.974
Educational level, *n* (%)				0.230
Primary school	14 (5.4)	12 (6.6)	2 (2.7)	
Lower-secondary school	113 (44.0)	76 (41.8)	37 (49.3)	
High-school/diploma	101 (39.3)	70 (38.5)	31 (41.3)	
University degree	29 (11.3)	24 (13.2)	5 (6.7)	
Work, *n* (%)				0.939
Employed	57 (21.8)	40 (21.7)	17 (22.1)	
Unemployed	140 (53.6)	97 (52.7)	43 (55.8)	
Student	11 (4.2)	8 (4.3)	3 (3.9)	
Retired	30 (11.5)	21 (11.4)	9 (11.7)	
Housewife/other	23 (8.8)	18 (9.8)	5 (6.5)	
Diagnosis, *n* (%)				0.132
Schizophrenia spectrum	186 (71.3)	127 (69.0)	59 (76.6)	
Bipolar disorder	57 (21.8)	46 (25.0)	11 (14.3)	
Personality disorders	18 (6.9)	11 (6.0)	7 (9.1)	
Age at onset (years)				0.069
<18	26 (10.0)	22 (12.0)	4 (5.2)	
18–30	129 (49.4)	97 (52.7)	32 (41.6)	
31–45	71 (27.2)	45 (24.5)	26 (33.8)	
46–60	31 (11.9)	18 (9.8)	13(16.9)	
≥61	4 (1.5)	2 (1.1)	2 (2.6)	
Illness duration (years)				**<0.001**
<1	50 (19.2)	22 (12.0)	28 (36.4)	
2–5	63 (24.1)	46 (25.0)	17 (22.1)	
6–10	43 (16.5)	35 (19.0)	8 (10.4)	
≥11	105 (40.2)	81 (44.0)	24 (31.2)	
Long-acting antipsychotics, *n* (%)				0.411
First-generation	73 (28.0)	49 (26.6)	24 (31.2)	
Risperidone	28 (10.7)	22 (12.0)	6 (7.8)	
Paliperidone	74 (28.4)	54 (29.3)	20 (26.0)	
Aripiprazole	74 (28.4)	53 (28.8)	21 (27.3)	
Olanzapine	12 (4.6)	6 (3.3)	6 (7.8)	
BPRS, mean (sd)	48.5 (14.1)	48.1 (14.1)	49.5 (14.0)	0.317
Kemp, mean (sd)	4.87 (1.49)	5.04 (1.47)	4.45 (1.45)	**0.004**
DAI-10, mean (sd)	2.28 (5.18)	2.88 (5.09)	0.83 (5.16)	**0.003**

Characters in bold indicate a *p* value <0.05. *BPRS* Brief Psychiatric Rating Scale, *DAI* drug attitude inventory, *n* number of patients, *sd* standard deviation.

### Survival analysis

**Fig. 2:**
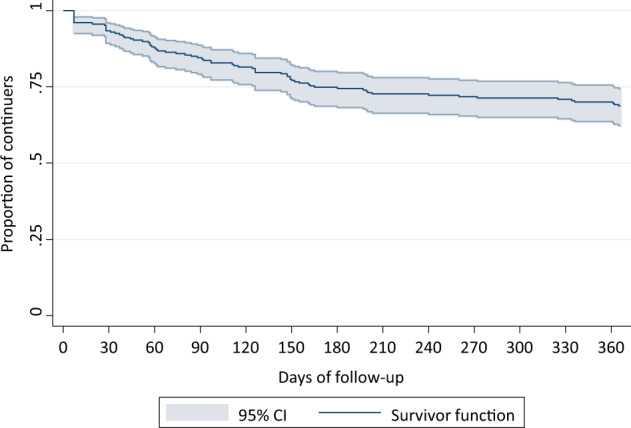
Kaplan–Meier survival estimate. Kaplan–Meier survival curve showing the proportion of continuers throughout the study follow-up. CI confidence interval.

Figure 2 shows the Kaplan–Meier estimates of survival rates in the whole sample. Of the 261 included participants, 77 (29.5%) discontinued the LAI prescribed over the course of follow-up (Figs. 1 and 2). Half of participants (52.1%) discontinued the treatment within the first trimester of the follow-up. The most common reasons for discontinuing included: adverse events (27.3%), switching to oral antipsychotics (24.7%), and non-adherence or refusal of the LAI (24.7%), while relatively few participants discontinued due to poor clinical benefit (3.9%) (Fig. 1).

### Mirror-image and pre-post analyses

The Brant test did not reject the proportional-odds assumption in any case (for the model with time only as predictor, *p* = 0.068 for the whole sample, *p* = 0.400 for continuers and *p* = 0.066 for discontinuers; for the model with continuer status and its interaction with time, *p* = 0.198), thus an ordered-logit regression was performed to predict the hospital admission intensity group. When comparing the year before and the year after the introduction of the LAI, the mirror-image analysis showed a statistically significant reduction of hospital admissions for continuers (*p* < 0.001), with a mean number of admissions decreased from 0.86 (standard error (se) 0.08) to 0.35 (se 0.06), while for discontinuers the reduction was non-significant (*p* = 0.059), from 0.79 (se 0.09) to 0.55 (se 0.12). In particular, continuers showed a greater increase in the proportion of participants with no hospital admissions (from 40.4% to 78.9%, while in discontinuers it increased from 40.0% to 64.5%) (Table 2). The BPRS mean score significantly decreased in both groups, and this trend was confirmed for each of the five subscales. Participants’ attitude towards medications as measured by the DAI-10 significantly improved in continuers (from 2.89 (se 0.37) to 4.86 (se 0.35), *p* < 0.001), but not in discontinuers (from 0.83 (se 0.58) to 1.55 (se 0.88), *p* = 0.389). Similarly, the Kemp registered a slight but statistically significant improvement of overall adherence as perceived by clinicians in continuers (from 5.04 (se 0.11) to 5.61 (se 0.09), *p* < 0.001), while this remained unchanged in discontinuers (from 4.45 (se 0.16) to 4.45 (se 0.31), *p* = 0.940).

**Table 2:** Mirror-image (hospitalization) and pre-post comparisons (all other outcomes).

	All participants			Continuers			Discontinuers		
	Baseline*** (Mean, SEM)	12 months*** (Mean, SEM)	*p* value of time	Baseline*** (Mean, SEM)	12 months*** (Mean, SEM)	*p* value of time	Baseline*** (Mean, SEM)	12 months*** (Mean, SEM)	*p* value of time
No. of hospital admissions (previous vs. current year)*	0.84 (0.06)	0.40 (0.06)	**<0.001**	0.86 (0.08)	0.35 (0.06)	**<0.001**	0.79 (0.09)	0.55 (0.12)	0.059
No. of days in hospital (previous vs. current year)**									
None	104/258 (40.31%)	175/233 (75.11%)	**<0.001**	74/183 (40.44%)	135/171 (78.95%)	**<0.001**	30/75 (40.00%)	40/62 (64.52%)	**0.020**
Up to 14 days	55/258 (21.32%)	22/233 (9.44%)		37/183 (20.22%)	16/171 (9.36%)		18/75 (24.00%)	6/62 (9.68%)	
More than 14 days	99/258 (37.37%)	36/233 (15.45%)		72/183 (39.34%)	20/171 (11.70%)		27/75 (36.00%)	16/62 (25.81%)	
BPRS*	48.52 (0.87)	35.45 (0.77)	**<0.001**	48.12 (1.04)	35.29 (0.86)	**<0.001**	49.47 (1.59)	35.94 (1.76)	**<0.001**
BPRS affect*	10.69 (0.27)	8.74 (0.23)	**<0.001**	10.55 (0.33)	8.72 (0.26)	**<0.001**	11.00 (0.47)	8.76 (0.53)	**<0.001**
BPRS positive*	11.59 (0.32)	7.36 (0.23)	**<0.001**	11.57 (0.39)	7.29 (0.26)	**<0.001**	11.62 (0.57)	7.61 (0.53)	**<0.001**
BPRS negative*	7.83 (0.23)	7.02 (0.21)	**0.002**	7.69 (0.26)	7.06 (0.23)	**0.021**	8.16 (0.48)	6.80 (0.50)	**0.040**
BPRS resistance*	9.27 (0.27)	6.00 (0.21)	**<0.001**	9.24 (0.33)	5.88 (0.22)	**<0.001**	9.34 (0.47)	6.45 (0.54)	**<0.001**
BPRS activation*	7.60 (0.21)	5.19 (0.16)	**<0.001**	7.59 (0.24)	5.22 (0.18)	**<0.001**	7.65 (0.39)	5.08 (0.34)	**<0.001**
Kemp*	4.87 (0.09)	5.34 (0.10)	**<0.001**	5.04 (0.11)	5.61 (0.09)	**<0.001**	4.45 (0.16)	4.45 (0.31)	0.940
DAI-10*	2.28 (0.32)	4.09 (0.34)	**<0.001**	2.89 (0.37)	4.86 (0.35)	**<0.001**	0.83 (0.58)	1.55 (0.88)	0.389
No. of antipsychotics (LAI + oral)*	1.06 (0.04)	1.15 (0.04)	0.079	1.05 (0.05)	1.36 (0.04)	**<0.001**	1.08 (0.07)	0.62 (0.07)	**<0.001**
Cumulative dose of antipsychotics (PDD/DDD)*	1.14 (0.11)	1.24 (0.07)	0.873	1.21 (0.16)	1.46 (0.08)	0.329	0.99 (0.10)	0.63 (0.09)	**<0.001**
Cumulative dose of all psychotropic drugs (PDD/DDD)*	1.91 (0.14)	1.73 (0.11)	0.240	2.07 (0.18)	2.12 (0.14)	0.832	1.51 (0.15)	0.82 (0.10)	**<0.001**
No. of adverse events*	0.46 (0.05)	1.35 (0.09)	**<0.001**	0.43 (0.06)	1.41 (0.10)	**<0.001**	0.52 (0.10)	0.96 (0.21)	0.053

Characters in bold indicate a *p* value <0.05. BPRS Brief Psychiatric Rating Scale, *DAI-10* Drug Attitude Inventory 10-items, *LAI* long-acting injective antipsychotics, *SEM* standard error of the mean, *PDD/DDD* ratio between prescribed daily dose and defined daily dose.

* Linear regression with individual random effects and robust standard errors.

** Odds Ratios from Ordinal Logistic regression with individual random effects and robust standard errors.

*** Maximum-Likelihood Estimates with Missing Values Estimates and robust standard errors was used for continuous variables.

In continuers, the total number of antipsychotics (including both LAIs and oral antipsychotics) significantly increased over the follow-up period, although their cumulative dose, as well as the overall cumulative dose of all psychotropic drugs, remained nearly unchanged. On the other hand, in discontinuers, the number of antipsychotics, their cumulative dose, and the cumulative dose of all psychotropic drugs significantly decreased over time (Table 2). The number of adverse events reported throughout the follow-up significantly increased only for continuers (Table 2).

### Differential improvement between continuers and discontinuers

When assessing the differential change over time between continuers and discontinuers, we found no differences in terms of clinical rating scale scores (*p* > 0.10 in all cases), while the other outcomes highlighted different trends between the two groups (Table 3). In particular, the group of continuers showed a higher probability of having less days hospitalized (Odds Ratio (OR) 0.38, 95% CI 0.16–0.93, *p* = 0.034), more adverse events (*p* = 0.039), more antipsychotics prescribed (*p* < 0.001), and a higher cumulative dose of both antipsychotics (*p* = 0.005) and all psychotropic drugs (*p* = 0.002).

**Table 3:** Interaction between time and being a continuer (differential improvement between continuers and discontinuers).

	Coefficient	Confidence interval	*p* value
No. of hospital admissions in the last year	−0.28	−0.58–0.02	0.070
Admission groups (no admission, admission up to 14 days, admission for more than 14 days)*	0.38	0.16–0.93	**0.034**
BPRS	0.74	−4.27–5.75	0.772
BPRS affect	0.36	−1.02–1.74	0.606
BPRS positive	−0.20	−1.86–1.47	0.818
BPRS negative	0.70	−0.71–2.11	0.330
BPRS resistance	−0.44	−1.89–1.01	0.551
BPRS activation	0.22	−0.86–1.30	0.685
Kemp	0.57	−0.12–1.25	0.107
DAI-10	1.28	−0.68–3.24	0.199
No. of adverse events in the last year	0.51	0.03–0.99	**0.039**
No. of antipsychotics (LAI + oral)	0.77	0.55–0.98	**<0.001**
Cumulative dose of all psychotropic drugs (PDD/DDD)	0.73	0.22–1.24	**0.005**
Cumulative dose of antipsychotics (PDD/DDD)	0.64	0.23–1.05	**0.002**

Characters in bold indicate a *p* value <0.05. *BPRS* Brief Psychiatric Rating Scale, *DAI-10* Drug Attitude Inventory 10-items, *LAI* long-acting injective antipsychotics, *PDD/DDD* ratio between prescribed daily dose and defined daily dose.

*Odds ratio from Ordered Logit Regression.

### Sensitivity analysis

Sensitivity analyses including only participants with schizophrenia spectrum disorders (186 participants) generally confirmed those performed on the global sample both in terms of statistical significance and of magnitude, although some differences arose when analyzing continuers and discontinuers separately. In this subset, the reduction in the number of hospital admissions and days hospitalized was statistically significant for both continuers and discontinuers. Further, there was no statistical differential change over time in terms of number of hospital admission and days hospitalized between continuers and discontinuers, while being a continuer was significantly associated with lower reduction of BPRS “affect” and “negative symptoms” subscales’ scores over time, and no significant differences emerged in terms of adverse events.

## Discussion

In this observational, longitudinal study, we performed mirror-image and pre-post analyses of 261 LAI-naive individuals with schizophrenia-spectrum disorders, bipolar disorders or personality disorders, and assessed whether LAI continuers and discontinuers had different clinical trajectories throughout the 12-month follow-up period. Although most demographic and clinical variables were broadly comparable between continuers and discontinuers at baseline, the former had significantly longer duration of disease, and better treatment adherence (as perceived by the clinician) and attitude towards medications (i.e., higher Kemp and DAI-10 scores). Interestingly, although overall symptoms significantly decreased over time for both continuers and discontinuers, the number of hospital admissions significantly decreased only for continuers, who also had a significantly larger decrease in the number of hospital days as compared to discontinuers. Similarly, treatment adherence (Kemp scale) and attitude towards treatments (DAI-10) significantly improved only for continuers. On the other hand, the cumulative number of antipsychotics and of medication-related adverse events showed a stronger increase in continuers as compared to discontinuers. Further, the dose of both antipsychotics and all psychotropic medications significantly decreased over time in discontinuers (while it remained nearly unchanged for continuers), arguably reflecting the negative attitude of these individuals towards psychotropic medications in general.

In general, these findings are in line with previous literature from observational studies showing that, compared to oral antipsychotics, LAIs might improve not only clinical outcomes (i.e., hospitalization, psychopathology)^5,16^, but also adherence and attitude towards treatments^35–37^. Our study confirms the benefits of LAIs for people with schizophrenia, expanding previous evidence to a larger group of clinical conditions (namely, bipolar disorders and personality disorders).

To our knowledge, this is the first observational study considering different clinical trajectories of LAI continuers and discontinuers over time. Among the mirror-image studies included in the meta-analysis by Kishimoto and colleagues^16^, one-third pooled together these two sub-populations and two-thirds analyzed only LAI continuers, ignoring the clinical outcomes of LAI discontinuers. We argue that data interpretation and clinical applicability might be notably biased in both cases.

This study suffers from several limitations. First, observational design prevents from drawing causal effects; therefore, findings should be regarded as merely associational and exploratory. Second, selecting LAI-naive individuals allowed a more homogeneous sample, although reduced the overall sample, penalizing statistical power. Third, we included individuals with different diagnoses aiming to be pragmatic and reflect real-world populations (including those prescribed off-label, which are rarely considered). This might have introduced heterogeneity, as different populations might respond differently to pharmacological treatments, and have different attitude towards medications. Although the subgroup analysis on people with schizophrenia-spectrum disorders generally confirmed the main findings, we could not perform additional subgroup analyses due to lack of statistical power. Fourth, we compared all LAIs together versus all oral antipsychotics together, and were not able to discern potential differential effects of single antipsychotics, again due to lack of statistical power. Finally, we measured the overall number of adverse events reported, but were not able to discern their impact on daily functioning and quality of life.

In terms of implications for clinical practice, our data further supports the notion that LAI formulations, as compared with oral antipsychotics, may offer added benefits to individuals with severe mental illness, although the risk of discontinuing the LAI over time should not be overlooked. However, added benefits may be relatively small in magnitude, and possibly counterweighed by higher cumulative doses of psychotropic medications and more adverse events. Therefore, a careful analysis of pros and cons of oral versus LAI formulations should be performed on a case-by-case basis at the beginning a throughout the course of the treatment, involving end users and their caregivers and family members into the decision-making process.

Further, clinicians need more accurate strategies to recognize individuals at risk of discontinuing LAIs, which might not be effectively detected by the sole routine clinical evaluation, consistently with previous literature suggesting clinicians’ tendency to underestimate adherence issues^38,39^. Pragmatically, employing a simple, self-administered, validated rating scale such as the DAI-10 might notably improve the ability to detect individuals at risk of discontinuing medications for various causes, including adherence issues.

As for research implications, future mirror-image and pre-post studies on LAI maintenance treatment should analyze LAI continuers and discontinuers separately, considering the different clinical trajectories of these two populations, and in view of the considerable proportion of LAI discontinuers, which accounted for more than one-fourth in the present study. Future studies are needed to provide better and more accurate estimates of the differences between these two populations, considering also the importance of health resource use and cost-effectiveness ratio.

## Supplementary information

Supplementary Material

## Data Availability

The dataset and relevant statistical syntaxes that were used to analyze data will be made available upon motivated request to the corresponding author.
