# Supplementary material for: Clinical trajectories of individuals with severe mental illness continuing and discontinuing long-acting antipsychotics: a one-year mirror-image analysis from the STAR Network Depot study
Source: Schizophrenia (Heidelb). 2023 Apr 17;9(1):23. doi: 10.1038/s41537-023-00342-3 (PMC10110527; doi:10.1038/s41537-023-00342-3)
Supplement: Supplementary file 1 — Supplementary Material [file 41537_2023_342_MOESM1_ESM.docx]

**Supplementary material**

**Clinical trajectories of individuals with severe mental illness continuing and discontinuing long-acting antipsychotics: a one-year mirror-image study (Ostuzzi et al.)**

**Index**

Additional details on methodology p. 2

Subgroup analysis: Survival analysis per diagnostic group p. 3

Sensitivity analysis: schizophrenia-spectrum subsample p. 4

**Additional details on methodology**

*Estimation of treatment duration*

Regression for interval-censored data implies the possibility to have some observations for which exact treatment duration is unknown, but only an interval with its possible values is available. In our case, patients leaving the treatment in the first week were assigned a treatment duration of 7 days, and all patients were assumed to have a treatment duration of at least 7 days. In case of discontinuation in an unknown date in the first semester or in an unknown semester, the minimum number of days was kept to 7, while in case of discontinuation in an unknown date in the second semester, the minimum number of days was calculated as the number of days in the first semester. In case of discontinuation in an unknown date in the first semester, the maximum value of days was calculated as the number of days in the first semester. In case of discontinuation in an unknown date in the second semester or in an unknown day in an unknown semester, the maximum value of days in the 12-month period (thus, either 365 or 366 days, depending on whether a leap year was crossed). In case of censoring at 6 months, the observation was excluded due to lack of 12-month follow-up data. All Kaplan-Meyer estimates are based on patients whose exact treatment duration is known.

**Subgroup analysis: Survival analysis per diagnostic group**

Figure 1S shows the Kaplan-Meier curves for each diagnostic group separately. To assess whether across-diagnosis differences in survival times are statistically significant, we performed a regression for interval-censored data on the whole sample, using diagnostic group as the only predictor. In particular, we performed a Gompertz regression with unrestricted ancillary parameter (let us define it *λ*) and chose the model based on its sign and statistical significance: an exponential model in case of lack of statistical significance while, in case of a statistically significant *λ* estimate*,* a Gompertz or a negative Gompertz model depending on its sign. Due to the negative and statistically significant value of the *λ* parameter (-0.005; p-value<0.001), a negative Gompertz model was performed. Differences in the harzard ratio across diagnostic groups were not statistically significant (p-value 0.115).

**Figure 1S. Kaplan-Meier survival estimates on each diagnostic group separately**

**Sensitivity analysis: schizophrenia-spectrum subsample**

Table 1S shows the analysis on the subset of people with schizophrenia-spectrum disorders, confirming improvement in clinical scales during the follow-up for the BPRS scale and its subscales (p 0.003 or lower in all cases) and Kemp (p 0.012) and DAI-10 scores (p < 0.001). Such results were confirmed in the group of continuers with the exception of BPRS negative subscales (where the decrease did not reach statistical significance); for discontinuers the improvement only took place for the BPRS scores as in the case of the whole sample, with DAI-10 and Kemp scales not seeing any improvement (even at the point-estimate level). We again found an increase in the number of adverse events for both groups (p-value < 0.01 in both cases) and in the number of LAI for continuers, that turned out to decrease for discontinuers, as well as their average cumulative dose of antipsychotic and of all psychotropic drugs (p-value 0.001 or lower in all cases). Both groups showed a statistically significant increase both in the number of adverse events (particularly large for continuers), and both in the average number of hospital admissions and in the percentage of patients with no admissions in the previous 12 months.

Table 2S shows a significantly stronger decrease for the BPRS Affect and BPRS Negative scales for discontinuers, change between continuers and discontinuers was found for clinical scales (p-value > 0.10 in all cases), while the different pattern seen both in the number of antipsychotic drugs and in their cumulative dose, as well in the cumulative dose of all psychotropic drugs, led to the statistical significance of the interaction parameter representing differential change (p-value = 0.001 or lower in all cases).

**Table 1S.** Mirror-image (hospitalization) and pre-post comparisons (all other outcomes).

|  | **ALL PARTICIPANTS** | | | **CONTINUERS** | | | **DISCONTINUERS** | | |
| --- | --- | --- | --- | --- | --- | --- | --- | --- | --- |
|  | **Baseline (Mean, SEM)** | **12 months (Mean, SEM)** | **p-value of time** | **Baseline (Mean, SEM)** | **12 months (Mean, SEM)** | **p-value of time** | **Baseline (Mean, SEM)** | **12 months (Mean, SEM)** | **p-value of time** |
| **N. of hospital admissions (previous vs. current year)*** | 0.74 (0.06) | 0.39 (0.07) | **<0.001** | 0.74 (0.08) | 0.37 (0.09) | **<0.001** | 0.73 (0.09) | 0.44 (0.11) | **0.031** |
| **N. of days in hospital**  **(previous vs. current year)**** |  |  |  |  |  |  |  |  |  |
| **None** | 77/183 (42.08%) | 124/162 (76.54%) | **<0.001** | 54/126 (42.86%) | 93/117 (79.49%) | **<0.001** | 23/57 (40.35%) | 31/45 (68.89%) | **0.009** |
| **Up to 14 days** | 40/183 (21.86%) | 16/162 (9.88%) |  | 28/126 (22.22%) | 12/117 (10.26%) |  | 12/57 (21.05%) | 4/45 (8.89%) |  |
| **More than 14 days** | 66/183 (36.07%) | 22/162 (13.58%) |  | 44/126 (34.92%) | 12/117 (10.26%) |  | 22/57 (38.60%) | 10/45 (22.22%) |  |
| **BPRS*** | 49.76 (1.00) | 36.88 (0.87) | **<0.001** | 49.39 (1.22) | 37.52 (0.97) | **<0.001** | 50.54 (1.74) | 34.74 (1.94) | **<0.001** |
| **BPRS Affect*** | 10.62 (0.33) | 8.90 (0.25) | **<0.001** | 10.45 (0.41) | 9.12 (0.29) | **0.001** | 10.98 (0.51) | 8.07 (0.51) | **<0.001** |
| **BPRS Positive*** | 12.18 (0.37) | 7.88 (0.28) | **<0.001** | 12.27 (0.45) | 7.98 (0.32) | **<0.001** | 12.00 (0.65) | 7.64 (0.58) | **<0.001** |
| **BPRS Negative*** | 8.40 (0.28) | 7.49 (0.24) | **0.003** | 8.14 (0.32) | 7.70 (0.27) | 0.169 | 8.97 (0.55) | 6.70 (0.52) | **0.001** |
| **BPRS Resistance*** | 9.49 (0.32) | 6.26 (0.24) | **<0.001** | 9.57 (0.39) | 6.30 (0.26) | **<0.001** | 9.32 (0.55) | 6.12 (0.56) | **<0.001** |
| **BPRS Activation*** | 7.47 (0.24) | 5.19 (0.19) | **<0.001** | 7.45 (0.28) | 5.29 (0.22) | **<0.001** | 7.53 (0.45) | 4.87 (0.38) | **<0.001** |
| **Kemp*** | 4.87 (0.11) | 5.22 (0.12) | **0.012** | 5.04 (0.13) | 5.48 (0.10) | **0.002** | 4.51 (0.18) | 4.44 (0.33) | 0.854 |
| **DAI-10*** | 1.76 (0.39) | 3.54 (0.42) | **<0.001** | 2.26 (0.48) | 4.40 (0.41) | **<0.001** | 0.71 (0.66) | 0.68 (1.11) | 0.976 |
| **N. of antipsychotics (LAI + oral APs)*** | 1.09 (0.04) | 1.15 (0.05) | 0.281 | 1.08 (0.05) | 1.40 (0.06) | **<0.001** | 1.10 (0.08) | 0.61 (0.07) | **<0.001** |
| **Cumulative dose of antipsychotics (PDD/DDD)*** | 1.14 (0.12) | 1.18 (0.08) | 0.726 | 1.16 (0.16) | 1.45 (0.10) | 0.125 | 1.08 (0.11) | 0.61 (0.10) | **0.001** |
| **Cumulative dose of all psychotropic drugs (PDD/DDD)*** | 1.74 (0.14) | 1.71 (0.13) | 0.879 | 1.84 (0.19) | 2.15 (0.18) | 0.134 | 1.52 (0.18) | 0.77 (0.11) | **<0.001** |
| **N. of adverse events*** | 0.49 (0.06) | 1.40 (0.10) | **<0.001** | 0.43 (0.07) | 1.45 (0.11) | **<0.001** | 0.63 (0.12) | 1.17 (0.16) | **0.007** |

Legend: BPRS=Brief Psychiatric Rating Scale; DAI-10=Drug Attitude Inventory 10-items; LAI=Long-Acting Injective antipsychotics; SEM=Standard Error of the Mean; PDD/DDD=ratio between Prescribed Daily Dose and Defined Daily Dose

* Linear regression with individual random effects, distinct variances between time points and robust standard errors

** Ordinal Logistic regression with individual random effects

*** Maximum Likelihood with Missing Values Estimates and robust standard errors was used for continuous variables

**Table 2S.** Interaction between time and being a continuer (differential improvement)

|  | **Coefficient** | **Confidence interval** | **p-value** |
| --- | --- | --- | --- |
| **N. of hospital admissions in the last year** | -0.09 | -0.41 to 0.23 | 0.589 |
| **Admission groups (no admission, admission up to 14 days, admission for more than 14 days*** | 0.58 | 0.22 to 1.56 | 0.284 |
| **BPRS** | 3.97 | -1.55 to 9.49 | 0.159 |
| **BPRS Affect** | **1.52** | **0.15 to 2.90** | **0.030** |
| **BPRS Positive** | 0.11 | -1.71 to 1.93 | 0.906 |
| **BPRS Negative** | **1.81** | **0.28 to 3.35** | **0.021** |
| **BPRS Resistance** | -0.08 | -1.82 to 1.65 | 0.926 |
| **BPRS Activation** | 0.53 | -0.72 to 1.78 | 0.406 |
| **Kemp** | 0.51 | -0.25 to 1.27 | 0.190 |
| **DAI-10** | 2.08 | -0.31 to 4.47 | 0.088 |
| **N. of adverse events in the last year** | 0.43 | -0.04 to 0.91 | 0.071 |
| **N. of antipsychotics (LAI + oral)** | **0.81** | **0.58 to 1.05** | **<0.001** |
| **Cumulative dose of all psychotropic drugs (PDD/DDD)** | **1.06** | **0.50 to 1.61** | **<0.001** |
| **Cumulative dose of antipsychotics (PDD/DDD)** | **0.75** | **0.29 to 1.21** | **0.001** |

Legend: BPRS=Brief Psychiatric Rating Scale to DAI-10=Drug Attitude Inventory 10-items to LAI=Long-Acting Injective antipsychotics to PDD/DDD=ratio between Prescribed Daily Dose and Defined Daily Dose

*Odds Ratio from Ordered Logit Regression
